# Pioneering Advances and Innovative Applications of Mesoporous Carriers for Mitochondria-Targeted Therapeutics

**DOI:** 10.3389/bjbs.2024.13707

**Published:** 2024-11-18

**Authors:** Mohamad Anas Al Tahan, Sana Al Tahan

**Affiliations:** ^1^ Aston Medical Research Institute, College of Health and Life Sciences, Aston University, Birmingham, United Kingdom; ^2^ Faculty of Pharmacy, Arab International University, Daraa, Syria

**Keywords:** mitochondria, targeting, porous carriers, mesoporous silica, TPP^+^

## Abstract

Mitochondria, known as the cell’s powerhouse, play a critical role in energy production, cellular maintenance, and stemness regulation in non-cancerous cells. Despite their importance, using drug delivery systems to target the mitochondria presents significant challenges due to several barriers, including cellular uptake limitations, enzymatic degradation, and the mitochondrial membranes themselves. Additionally, barriers in the organs to be targetted, along with extracellular barriers formed by physiological processes such as the reticuloendothelial system, contribute to the rapid elimination of nanoparticles designed for mitochondrial-based drug delivery. Overcoming these challenges has led to the development of various strategies, such as molecular targeting using cell-penetrating peptides, genomic editing, and nanoparticle-based systems, including porous carriers, liposomes, micelles, and Mito-Porters. Porous carriers stand out as particularly promising candidates as drug delivery systems for targeting the mitochondria due to their large pore size, surface area, and ease of functionalisation. Depending on the pore size, they can be classified as micro-, meso-, or macroporous and are either ordered or non-ordered based on both size and pore uniformity. Several methods are employed to target the mitochondria using porous carriers, such as surface modifications with polyethylene glycol (PEG), incorporation of targeting ligands like triphenylphosphonium, and capping the pores with gold nanoparticles or chitosan to enable controlled and triggered drug delivery. Photodynamic therapy is another approach, where drug-loaded porous carriers generate reactive oxygen species (ROS) to enhance mitochondrial targeting. Further advancements have been made in the form of functionalised porous silica and carbon nanoparticles, which have demonstrated potential for effective drug delivery to mitochondria. This review highlights the various approaches that utilise porous carriers, specifically focusing on silica-based systems, as efficient vehicles for targeting mitochondria, paving the way for improved drug delivery strategies in mitochondrial therapies.

## Introduction

### Mitochondria and Its Importance in Biology

The mitochondria are energy-producing organelles that are present in aerobic organisms while being the primary source of reactive oxygen and nitrogen species (RONS), and possessing the ability to produce adenosine triphosphate (ATP) from the conserved redox energy of nutrients [1]. The mitochondria are usually termed the “powerhouse of the cell” mainly due to its production of ATP, and their importance for cell proliferation and survival, as well as their support to anabolism features through the generation of tricarboxylic acid (TCA) metabolites. Two of the generated metabolites are oxaloacetate and citrate, which in turn generate nucleotides and lipids, respectively [2]. The mitochondria are involved in both cell death and survival, as well as the progression of numerous diseases that include: neurodegeneration, genetic disorders, cancer, and diabetes. This is due to their crucial role in the function, regulation, and influencing the redox status, as well as signalling ion homeostasis [3]. Furthermore, it has been reported that the mitochondria are responsible for generating 90% of the intracellular reactive oxygen species (ROS) and high levels of H_2_O_2_, making them surrounded in high ROS environments. Yet, the high abnormal biochemical factors with excess ROS production affect several body moieties, causing irreversible oxidative damage to DNA, lipids, and proteins [4]. In addition, the mitochondria significantly influence cell ageing, as their functional decline becomes evident with age. This decline is linked to several factors, including mitochondrial DNA mutations, increased oxidative stress, diminished energy conversion efficiency, and the formation of danger-associated molecular patterns (DAMPs), which play a critical role in triggering inflammatory and immune responses, contributing further to cellular ageing and degeneration [5].

### Mitochondrial Targeting for Enhanced Drug Efficacy

The mitochondria play a crucial role in energy production [6], cellular metabolism, and cell death regulation [7], making them ideal targets to enhance drug efficacy, particularly in cancer treatment [8]. In tumour tissues, the mitochondria can alter their metabolic phenotypes to adapt to the high demands for energy and macromolecular synthesis [9]. Additionally, they can interact with the tumour microenvironment, receiving signals from cancer-associated fibroblasts that influence mitochondrial function. Cancer cells may also adopt a hybrid metabolic phenotype, enabling them to utilise both glycolysis and oxidative phosphorylation [10]. By targetting the mitochondria, therapies can disrupt cancer cell metabolism by depriving cells of the necessary metabolites for macromolecule synthesis and producing oncometabolites [11], induce apoptosis via the mitochondrial outer membrane permeabilisation that is a critical event in the process of regulated cell death [12], and improve therapeutic outcomes while minimising side effects [13]. Further, the mitochondria can affect bone remodelling through influencing protein function and affecting stem cell mobilisation, proliferation, differentiation, and inflammation [14]. Therefore, developing novel drug carriers that can reduce ROS enhances the treatment of oxidative stress-related diseases and tissue regeneration [15]. Over recent decades, significant advances in mitochondrial biology have paved the way for the development of mitochondria-targeted therapies. This approach offers a promising strategy for improving drug efficiency, especially for diseases where mitochondrial dysfunction is a key factor, providing more precise and effective treatments for patients [16, 17].

Multiple candidates have been researched and utilised as moieties for targeting the mitochondria. One example is MitoQ, an antioxidant capable of crossing the blood-brain barrier, which influences mitochondrial respiration by creating a pseudo-mitochondrial membrane potential [18]. Another example is SkQ1, another antioxidant that can penetrate membranes when linked to positively charged cations such as Triphenylphosphonium (TPP+), allowing it to accumulate up to a thousand folds within the inner layer of the inner mitochondrial membrane [19]. Other drugs were reported including Elamipretide (SS-31), a small peptide that targets the inner mitochondrial membrane and is designed to enhance mitochondrial function in aging. It has been shown to be well-tolerated and beneficial for patients with heart failure [20, 21]. The mitochondrial targeting effect is based on Elamipretide binding to cardiolipin on the inner mitochondrial membrane through electrostatic and hydrophobic interactions. This binding stabilises cardiolipin and prevents it from converting cytochrome c into a peroxidase, preserving its electron transport role and enhancing mitochondrial function. By supporting cardiolipin, SS-31 protects mitochondrial cristae structure, promotes oxidative phosphorylation, and reduces oxidative stress, which helps maintain energy production and cellular health, especially in age-related mitochondrial decline [22, 23].

### Barriers to Mitochondria Targeting

Considering the importance of mitochondria throughout the system’s biology, targeting it has been considered an approach for enhancing drug delivery and treatment. Targeting the mitochondria faces numerous barriers related to the physiology of the body, such as insufficient cellular uptake, lysosomal degradation, and mitochondrial membranes, which highly affect target efficiency. In addition, the targeted organs could impose a barrier to drug delivery, considering that they contain a barrier since these tissue microenvironments possess different physiological conditions, such as elevated pH values and higher enzymatic activity [24, 25].

The extracellular barriers include the reticuloendothelial system (RES) which contains cellular and non-cellular components including the phagocytes, working towards clearing particles that are larger than 200 nm, and eliminating the circulating nanoparticles after systematic administration [26, 27]. Additionally, the tissue stroma, extracellular matrix and connective tissue cells present additional barriers to the remaining nanoparticles that escaped RES and are directed towards the mitochondria [28]. As for the intracellular barriers, they include the endosomes that entrap the nanoparticles after internalisation. Further, the mitochondrial membranes create additional barriers as the mitochondria contain the outer mitochondrial membrane (OMM) and inner mitochondrial membrane (IMM). The OMM is porous in structure and surrounds the organelles, while on the other hand, the IMM is compact and hydrophobic, folds and enhances the mitochondrial surface area. Furthermore, both membranes control access, as molecules below 5,000 Da can diffuse, while CO_2_, oxygen and water can pass through the OMM and IMM, respectively [2, 24].

According to Pegoraro et al., the design of a successful targeting drug delivery system (DDS) should focus on four aspects: the size, shape, and rigidity of the drug carrier, as they dictate cellular uptake, surface area, and diffusion. The last and most crucial factor for mitochondria is the charge, considering the latter’s negative charge, in which positively charged moieties will favour better interaction [25].

### Mitochondria Targeting Approaches

As mentioned above, the mitochondria play crucial roles in system biology and affect numerous processes such as energy production and cell ageing. Additionally, it has been reported that the mitochondria regulate the stemness of non-malignant stem cells, and that altering the mitochondrial function dictates the acquisition and maintenance of stemness of non-cancerous cells [29]. In general, cationic moieties with lipophilic properties are considered an effective approach to target the mitochondria based on the electrostatic interactions between them (the cationic moieties) and the anionic inner membrane of the mitochondria. Yet, the rapid clearance and the non-specific tissue distribution of the cationic moieties restrict their *In vivo* applications [30, 31].

**FIGURE 1:**
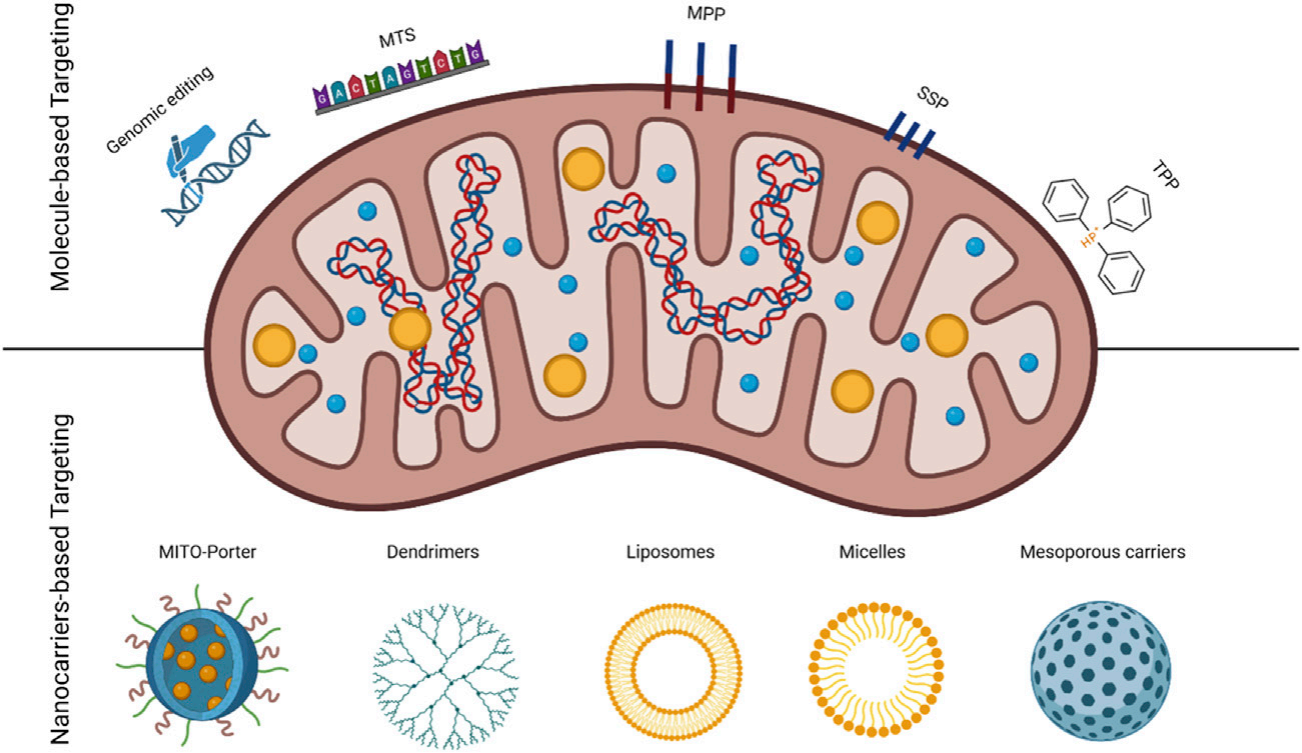
Schematic of mitochondria identifying two targeting approaches; molecule and nanoparticle-based with the used carriers.

There are several approaches to target the mitochondria, which can be classified as molecule- and nanocarrier-based targeting. The two approaches are highlighted in Figure 1 and explained in the following sections.

### Molecule-Based Drug Delivery Systems for Mitochondria Targeting

Mitochondrial genome editing is a novel proposed technique that has been discovered in the past two decades, where specific DNA sequences in the mitochondria could be targeted and modified using imported gene-editing proteins. In animal models and heteroplasmic cells, this can occur through specifically cleaving and eliminating mutant mitochondrial DNA via mitochondria-targeted nucleases [32]. One of the reported techniques of genomic editing is the use of clustered regularly interspaced short palindromic repeats-associated protein-9 nuclease (CRISPR-Cas9), which could have the ability to perform mitochondrial DNA gene editing [33]. However, this approach is still in its early stages and requires further development due to several challenges associated with CRISPR-Cas9, such as the potential for unintended genetic modifications and disruption of regulatory elements [34]. Another possible technique is the use of mitochondrial targeting sequences (MTS), possessing little sequence similarity to the mitochondrial proteins’ sequence since they have an amphiphilic α-helic, but consisting of 15–55 amino acids. These sequences could target the OMM, IMM, or the intermembrane space (IMS) to deliver proteins to the matrix, making them possible candidates as mitochondria-targeting DDSs [35–37].

Mitochondrial penetrating peptides (MPPs) are modified cell-penetrating peptides (CPPs), which have the ability to penetrate the mitochondrial membranes. They could be positively charged, which enhances their interaction with negatively charged mitochondria. Also, they could include lipophilic amino acids to enhance the penetration and interaction with the IMM, considering the latter’s higher lipophilicity [38]. Szeto–Schiller peptides (SSPs) are another variation of penetrating short peptides where they are composed of a small number of amino acids [4–10], while possessing a D amino acid and alternating cationic and aromatic amino acids [39].

Triphenylphosphonium (TPP+) is one of the most common mitochondria-targeting moieties due to several characteristics: large ionic radius, high lipophilicity, and the diffusion of the positive charge on three phenyl rings. These properties provide TPP+ with distinctive features that allow it to move freely across biological membranes without transporters and accumulate inside the mitochondria. This accumulation is due to the membrane potential and the difference in charge, which could reach 100–500 folds of higher accumulation for every 60 mV of membrane potential [40–42].

### Nanocarrier-Based Drug Delivery Systems for Mitochondria Targeting

Mito-porters are liposome-based drug carriers that carry the drug cargo and deliver it to the mitochondria through a membrane fusion mechanism. The surface can be functionalised to enhance the interaction since the modified drug carriers are internalised via micropinocytosis compared to the clathrin-mediated endocytosis for cationic liposomes [43]. The drug delivery process using these carriers follows several consecutive steps: the internalisation followed by effective nedosomal escape, the electrostatic interaction with the mitochondrial membrane, and the membrane fusion to deliver and release the loaded drug cargo [44].

Dendrimers are polymeric-based nanoparticles that possess a very distinctive feature: being highly branched in three dimensions while preserving symmetry. They are considered good candidates as DDSs for targeting due to their monodispersity, controlled size, and abundant functional groups on the surface, making them suitable for encapsulation [45]. Amine-functionalised cationic dendrimers could condense the genetic materials like plasmid DNA into nanoparticles while protecting them against degradation. Additionally, they can be functionalised with targeting ligands such as TPP^+^, presenting them with enhanced targeting properties [46].

Liposomes are spherically shaped nanoparticles made from two layers of phospholipids, where they enfold an aqueous core while the hydrophobic region is in the space between the two lipid bilayers [47]. Lipsosomes have great potential for targeting the mitochondria if they are functionalised with numerous ligands, proteins, and moieties that have an affinity to the mitochondria and, thus, enhance targeting capabilities. They can be used to manage breast cancer and overcome multi-drug resistance by loading the active compound (paclitaxel), which will accumulate inside the cancerous cells and facilitate mitochondrial-based targeting. Further, they can be modified with TPP^+^, which enhances the interaction due to its cationic features [48, 49].

Micelles are amphiphilic copolymers consisting of an external hydrophilic shell and internal lipophilic core suitable for encapsulating poorly soluble drugs to provide an easy functionalisation for targeting purposes [50]. Micelles have been used to design pH/ROS-responsive DDS of doxorubicin (DOX) to target the mitochondria, while PEG and TPP^+^ have been used to enhance targeting. Another approach utilised chondroitin sulfate to shield functionalised micelles, which included poly(d,l-lactide) (PLA) in addition to the TPP^+^-PEG [51, 52].

The use of mesoporous silica carriers is the main aim of this review and will be discussed in the following sections.

### Porous Carriers Classification

Generally, conventional particles for drug delivery are spherically shaped and possess a variety of morphologies. However, porous carriers, conversely, can also be spherical but have several holes within the structure that provide the loading carriers with an initial burst release of the drug [53]. The pores within these carriers can either have an ordered pore arrangement and uniform size or have a broad size distribution with a disordered pore size [54, 55]. According to the International Union of Pure and Applied Chemistry (IUPAC), porous materials are classified into three categories depending on the pore size: microporous where the pore size is less than 2 nm, mesoporous where the pore size is in the range of 2–50 nm, and macroporous where the pore size exceeds 50 nm [56].

**FIGURE 2:**
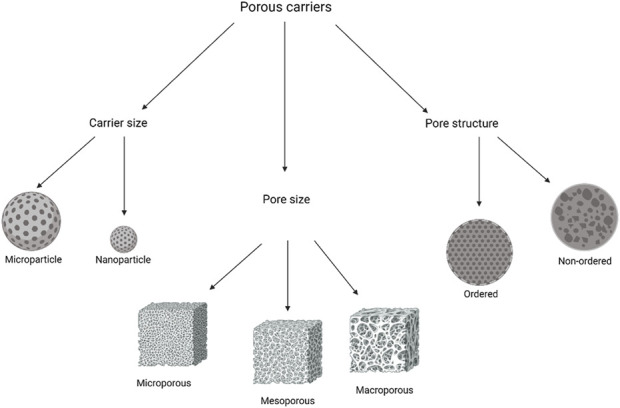
Porous carriers classification based on carrier size, pore size, and pore arrangement.

The mesoporous carriers have gained significant attention in research, and they are categorised into silicon-based and non-siliceous mesoporous materials. The first are inorganic materials and the most studied as they are synthesised from the condensation of either silicon alkoxides or sodium silicates around a template, which is an ordered surfactant [57]. On the other hand, the synthesis of non-siliceous mesoporous materials can be achieved from polymers, carbon nitrides, carbons, and metals [58]. The different classifications of porous carriers are identified in Figure 2.

Mesoporous silica were first developed in Japan by Yanagisawa et al. in the 1990s and was industrially produced by Mobil Corporation Laboratories [59]. There are several families of mesoporous silica, and amongst the most famous is the Molecular 41 Sieves (M41S) family, which contains several members, including MCM-41, MCM-48, and MCM-50, as the MCM stands for Mobil Crystalline Material [60]. Many types have been subsequently developed, and among the most reported in the literature is Santa Barbara 15 (SBA 15), a nanoparticle form of mesoporous silica that was invented by a research group in the United States [61, 62]. Mesoporous silica were originally designed for chemistry applications, notably in analytical and electrochemical fields. Its high surface area, consistent pore size, and adaptable structure made it ideal for catalysis, adsorption, and sensing tasks [63]. The chemical stability of SiO_2_ matrices makes the abundant pores in mesoporous silica ideal for accommodating a wide range of molecules. Additionally, the exceptional biocompatibility of SiO_2_ enhances its potential for various applications, that include drug delivery systems, bioimaging, and biosensing, where safe and effective interaction with biological systems is crucial [64]. However, the first use of mesoporous silica as carriers for drug delivery was reported in 2001, when they were explored as potential drug carriers for Ibuprofen to study the drug’s release behaviour from these porous structures. This marked the beginning of mesoporous silica’s application in controlled drug delivery [65].

Mesoporous silica nanoparticles (MSNs) have many characteristics that make them sought after as drug delivery carriers. For instance, the particle and pore sizes can be fine-tuned to be between 2 and 50 nm, which allows the carrier to contain molecules of varying sizes within the porous structure [66]. Also, MSNs possess a high surface area that could range between 600 and 1,000 m^2^/g, which makes the carrier a great candidate for loading purposes. Furthermore, this high surface area provides the opportunity for functionalisation, where the carrier’s surface is linked with different moieties that enhance targeting, enable controlled release, and determine interactions with the loaded molecules, as well as cells and tissues [67]. In addition, MSNs can be functionalised with gatekeepers (capping materials or molecular gates) that allow the delivery of the loaded drug to be triggered by applying a stimulus. The inclusion of the gatekeepers assists in retaining the entrapped molecules within the porous structure, which are to be delivered in the presence of an external stimulus. The use of this approach provides an alternative for liposomes or polymers in controlled release, as the previous two release their drug cargo based on diffusion or the carrier’s decomposition [68]. The release of the loaded drug cargo, as mentioned above, is related to stimuli, where it can be either internal or external, according to Mekaru et al. [69]. The internal or intracellular stimuli could be the activation by low pH, redox, and biomolecules or enzymes. On the other hand, external stimulants could be light or magnetic fields. Following the synthesis of MSNs in the 1990s and the first medical application for drug delivery in 2001 for the delivery of ibuprofen, they have attracted the attention of scientists for different uses and applications [65].

Compared to other carriers, and especially liposomes, MSNs offer several advantages. They exhibit stability in non-aqueous solutions, enabling the loading of hydrophobic drugs in organic solvents. Additionally, their large, easily functionalised surface area allows hydrophobic drugs to be loaded even in aqueous environments, as the surface serves as an effective reservoir for the drug [70]. Additionally, MSNs offer superior stability in biological environments compared to other nanoparticles, providing robust protection for encapsulated drugs. The rigid silica framework serves as a barrier against enzymatic degradation, which is particularly critical for sensitive therapeutics such as proteins and peptides that are vulnerable to breakdown in complex biological fluids. Furthermore, mesoporous carriers can be effectively used to load proteins for oral drug delivery, shielding them from enzymatic degradation, protecting them from pH variations in the gastrointestinal tract, and ultimately enhancing their bioavailability. This dual protection ensures that the therapeutic agents remain intact until they reach their target site, improving treatment efficacy [71, 72].

### Mesoporous Silica-Based Drug Delivery Systems for Mitochondria Targeting

There are different approaches for using MSNs as DDSs to target the mitochondria. This could be passive, depending on the characteristics of diseased tissues, or active, which focuses on functionalising the carrier and the use of different ligands, and magnetic-based that includes implementing an external magnetic field to draw the nanoparticles (NPs) to inflamed tissues or disease sites [73]. Additionally, stimuli-responsive-based targeting can be included in this category as the MSNs will release their loaded drug cargo in response to certain stimuli, which can be divided into internal and external stimuli-responsive action according to Colilla and Vallet-Regi [74].

For passive targeting, MSNs could accumulate inside targeted tissues, whether tumours or inflammatory areas, via two pathways. They include the enhanced permeability and retention (EPR) effect and the extravasation through leaky vasculature and the subsequent inflammatory cell-mediated sequestration (ELVIS) mechanisms in tumours and inflammatory tissues, respectively. As for active targeting, it involves the addition of targeting molecules to the surface of MSNs to selectively target intended cell types without harming other tissues. Amongst the most employed targeting moieties are aptamers, peptides, and proteins [75, 76].

**FIGURE 3:**
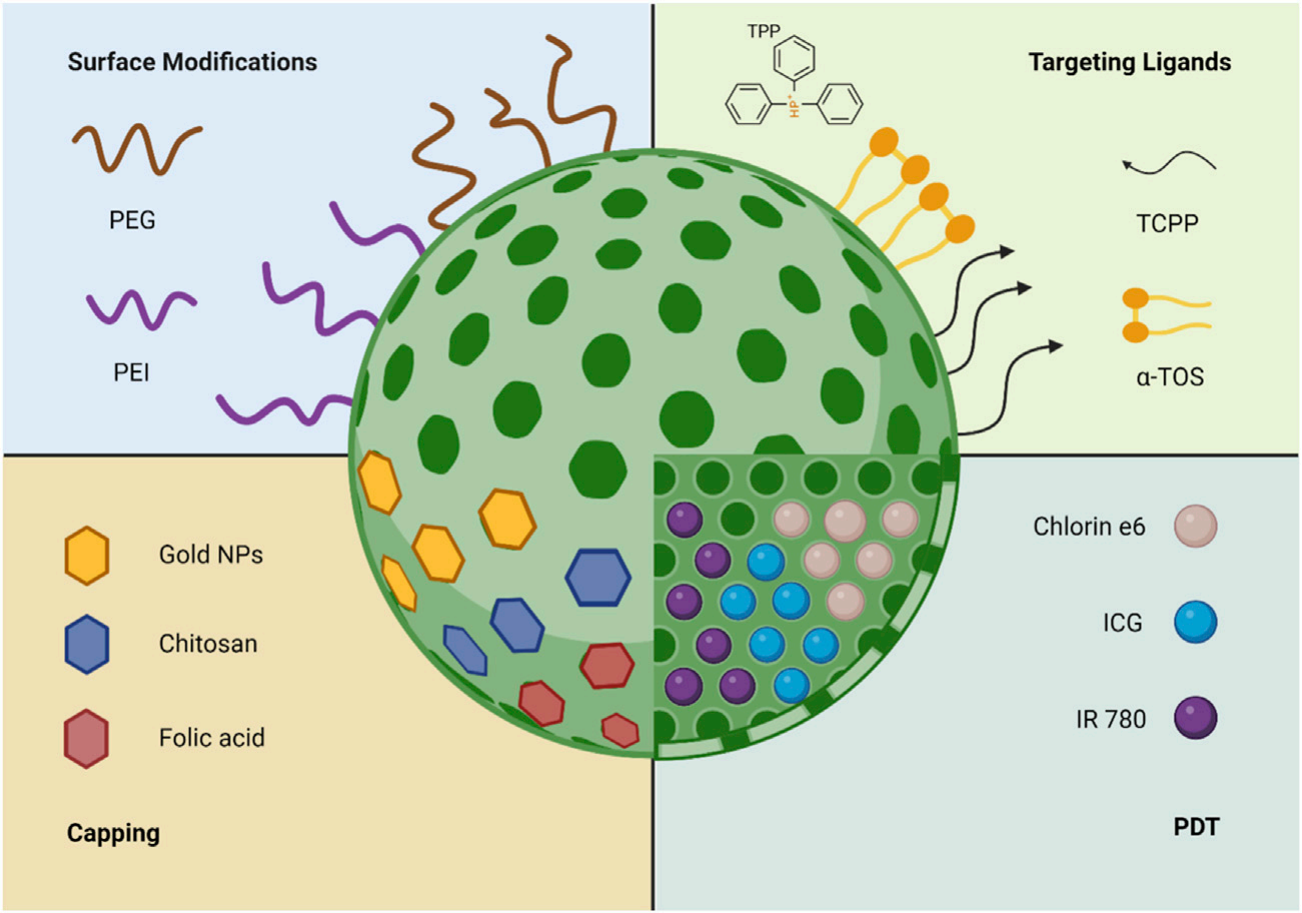
The different uses and functionalisation methods of MSNs for mitochondria targeting.

Recently, the use of mitochondrion medicine has emerged as a biomedical direction for treatment, focusing on the mitochondria as the therapy target for numerous diseases, including cancer. Additionally, as mentioned above, the physiological role of the mitochondria in regard to programmed cell death and energy production is the critical concept for targeting it [77]. As reported earlier, MSNs can be used as targeting DDSs due to their small size and easily functionalised surface. Yet, an additional advantage is their accumulation within the targeted tissues, which enhances the precision of drug delivery and minimises side effects [78]. Interestingly, the size of MSNs provides an additional advantage as small-sized MSNs interact with cells without disorganising the membrane [79]. For this reason, research has focused on using MSNs as possible carriers for mitochondrial-based targeting. The following sections will focus on the different uses of MSNs as well as numerous functionalisation approaches, which include using targeting moieties like TPP^+^, surface modification, photodynamic therapy (PDT), and capping. These applications are summarised in Figure 3.

The use of TPP^+^ as a targeting moiety with MSNs has been investigated due to TPP^+^’s hydrophobic properties, positive charge, and the ability to accumulate inside the negatively charged mitochondria based on electrostatic interactions [44]. Interestingly, using mesoporous silica has been reported to construct a liposomal-based nano platform for mitochondrial targeting [80]. This work used MSNs as the core for the developed DDS, where TPP^+^ was attached on the surface, with subsequent modification in other moieties. The inclusion of MSNs has enhanced the mechanical stability of liposomes to withstand long circulation *In vivo* and avoid deformation as the modification of TPP^+^ can take many forms to enhance its therapeutic and targeting effects. Additionally, TPP^+^ surface functionalised-MSNs were loaded with DOX and β-Lapachone, which has the ability to respond to endogenous reactive oxygen species (ROS) in cells and increase the levels in cancerous cells *in situ*. Furthermore, this approach utilised chitosan coating for TPP^+^, which has a higher biocompatibility, biodegradability, and enhances the stability of the nanocarriers while maintaining mitochondrial targeting properties [81]. Another example explored the application of TPP^+^-functionalised MSNs for delivering α-Tocopheryl succinate (α-TOS), an effective therapeutical mitochondrial agent that induces apoptosis, which focused on using TPP^+^ as a targeting ligand as well as α-TOS to achieve good targeting efficiency [82]. The results presented high cellular uptake and low cytotoxicity against HeLa and HepG2 cells. In addition, the internalisation of MSNs via endocytosis in mammalian cells is a critical factor for the use of these carriers for targeting purposes. The inclusion of TPP^+^ as a targeting ligand has also been reported as a DDS for DOX, where TPP^+^ provided the targeting properties and DOX presented the anti-cancerous effects [83]. This approach resulted in an enhanced cancer cell-killing efficiency in HeLa cells, as well as a decrease in ATP production and a decreased mitochondria membrane potential. Another example of delivering DOX has been reported by Naz et al [84]. Their work focused on delivering DOX via hyaluronic acid (HA) and TPP^+^-modified MSNs. In this formulation, HA had a dual action in which it acted as a capping agent for the pores, and as a targeting ligand due to its degradation by hyaluronidase in the acidic environment of cancerous tissues. The novel carrier exhibited a selective uptake by CD44 receptor-mediated endocytosis and accumulated in the mitochondria while releasing DOX due to HA’s degradation.

An additional example of using TPP^+^ was reported by Cheng et al. in 2016. Their work focused on utilising MSNs to encapsulate DOX, while the surface of silica particles was functionalised using tumour-targeting cellular membrane-penetrating peptide (TCPP) and TPP^+^ to seal the pores via disulfide bonds. This platform presented a synergistic effect to target cancer cells and penetrate cell membranes to release the anti-cancer drug and mitochondria-targeting moiety in the cytoplasm [85].

#### Photodynamic Therapy

There are several approaches to mitochondrion-medicine, and among these is photodynamic therapy (PDT). The process utilises photosensitisers that are able to locally generate cytotoxic ROS, which is preceded by deactivating the mitochondria to ensure the release of adequate ROS [86]. Additionally, PDT can induce immunogenic cell death in tumour cells by targeting the mitochondria, which plays a crucial role in initiating the immune response against the cancer cells [87]. The following section will focus on several attempts to use MSNs for PDT-based mitochondrion medicine.

In 2022, Wang et al. designed a novel mesoporous silica nanospheres-based platform for targeting the mitochondria that was based on PDT. Their approach was based on generating ROS in mitochondria under near-infrared (NIR) irradiation, which activated mitochondrial apoptosis pathways. The mesoporous nanospheres were chosen as the carrier’s core and loaded with indole-3-carbinol and geldanamycin, which synergistically affected the activation of apoptosis pathways in mitochondria. The nanospheres were coated with MnO_2_ nanoparticles that acted as gatekeepers, which were subsequently destroyed in the presence of acids in the microenvironment of tumour cells, releasing the drug. This led to an enhanced PDT effects and activated the mitochondrial apoptotic pathways [88]. Another example that utilises the concept of PDT has been reported by Tian et al. Their work comprised loading MSNs with IR780 (mitochondria targeting moiety) and lactate oxidase as specific-targeting metabolism nanomodulators. The combination of these two components inhibited tumour metastasis by disrupting mitochondrial function and depriving the cells of lactate. The photodynamic effects of IR780 induced a photodynamic starvation effect, intensified the hypoxic state within cancerous cells, and enhanced the overall therapeutic impact. The new developed carrier also possessed photodynamic and photothermal abilities for mitochondrial damage [89].

Furthermore, another article has reported the development of PDT-based MSNs for mitochondria targeting [90]. This work utilised chlorin e6, a molecule photosensitiser and attached it covalently to both the internal and external surfaces of MSNs. Subsequently, TPP^+^ was added to provide mitochondria-targeting properties, and upon laser irradiation, large amounts of ROS were generated from chlorin e6, which caused cell apoptosis.

Another variation of MSNs-TPP^+^ has been proposed by Shi et al. who reported using hollow MSNs with a visible core suitable for loading large quantities. This concept has been explored as an NIR-triggered carrier for mitochondria targeting [91]. This approach used TPP^+^ as a mitochondria targeting moiety with DOX and indocyanine green (ICG) as encapsulated drug cargo and L-menthol as the gatekeeper. ICG is a biocompatible NIR photosensitiser that possesses both photodynamic and photothermal activities by producing ROS and elevating the temperature of the cells, respectively. This dual action proved helpful for mitochondria targeting after the addition of TPP^+^. L-menthol was added as a gatekeeper since it had a reversible solid-liquid transition, making it a good candidate for entrapping the active pharmaceutical ingredient (API).

#### Surface Modification

Due to the small size of MSNs, they might aggregate, and to overcome this, polyethylene glycol (PEG) was added to decrease the aggregation occurrence and prolong MSNs’ circulation upon administration. This also helped the MSNs to avoid being collected by the RES in the liver and spleen as well as to evade phagocytosis [75].

Lin et al. have designed a nanocarrier composed of PEGylated bilayer-MSNs for the drug delivery of paclitaxel and curcumin. They used PEG, considering its properties in weakening the clearance effects of the liver and spleen and causing a long drug circulation in the body. Their new carrier has exhibited longer circulation time in compassion to control carriers that did not contain PEG. Moreover, it had shown that the subcellular localisation was mainly in the mitochondria, showcasing good targeting characteristics for breast cancer [92, 93]. Another example of the use of PEGylated lipid MSNs was based on the work of Choi et al. in 2016. This work focused on the co-delivery of Axitinib and Celastrol (an anti-cancer drug that induces the inhibition of HIF-1α) to target angiogenesis and mitochondrial-based apoptosis in cancer. Their approach was based on loading Celastrol and Axitinib into the MSNs and the PEGylated bilayer, respectively. Their results revealed that this combination had provided a synergistic therapeutic effect and was apoptotic against cancer cells, where Axitinib was released first, followed by Celastrol [94].

In 2018, Choi et al. reported the use of MSNs for the controlled drug delivery and mitochondria targeting via celastrol [95]. Their approach included the PEGylation of MSNs to prolong their blood circulation and reduce their uptake. Additionally, the inclusion of poly L-aspartic acid (PLD) inversed the cationic charge of the carrier to increase the drug release at acidic values (pH: 5) when the carboxylic groups were protonated. This carrier inhibited the expression of the apoptosis protein HIF-1α in mice.

#### Capping

Due to the porous structure of MSNs, they might release the loaded drug cargo before reaching the target site. Therefore, capping materials are included to maintain the loaded drug cargo and release it upon interaction with a stimulus [96]. The following section focuses on the use of capped MSNs for mitochondrial-based therapy.

For instance, Bhavsar et al. reported the use of dual-responsive MSNs for breast cancer-targeted therapy [97]. Their work used chitosan and folate as capping materials for the pores after loading with DOX, while cystamine was used to provide the redox-based stimuli for targeting. The inclusion of the previous three materials has offered both redox and pH-responsive targeting properties for the MSNs-based carrier. MSNs can be functionalised with numerous moieties to achieve efficient targeting. For example, DOX-loaded MSNs were modified with dendritic polyglycerol while containing tariquidar (P-gp inhibitor), for the treatment of multi-drug resistance of breast cancer stem cells [98]. The use of dendritic polyglycerol had a dual action as it capped the pores of MSNs and as a pH-responsive material to achieve intracellular drug release.

Using gold nanoparticles as gatekeepers is another form of MSNs capping functionalisation for mitochondria targeting [99]. This work used amino-functionalised MSNs while being capped with intracellular glutathione-bound gold nanoparticles. Upon intracellular binding to glutathione, the nanoparticles induced oxidative stress that weakened the cancerous cells and made them more susceptible to chemotherapy, due to the effective redox triggering of glutathione. Furthermore, the literature has described the development of a redox-responsive MSN-gold nanoparticles as DDSs for tumours [100]. This work focused on loading a synthetic anti-cancer compound J1 via phenylboronic acid (PBA)-equipped MSNs and pore blocking using gold nanoparticles. PBA was used for tumour targeting, while gold nanoparticles provided surface functionalisation with Au-N groups that were broken down and interchanged with sulphur. This novel carrier caused mitochondrial-dependant apoptosis in MCF-7 cells due to oxidative stress while exhibiting a higher therapeutic effect due to increased cellular internalisation.

#### MSNs Modifications

Modifications to MSNs can take many forms, such as developing a bionic MSN-based nanocarrier for tumour treatment with mitochondria-targeting properties [101]. This carrier was composed of combretastatin A4 phosphate and vitamin K_2_ as the main API that was responsible for mitochondria dysfunction and vasculature destruction. Additionally, the bionic nanocarrier contained several agents that served as artificial affinity reagents for vascular endothelial growth factors to inhibit angiogenesis. This approach presented an enhanced therapeutic activity and mitochondria defunctionalisation, where multimodal starvation therapy was achieved.

In addition, MSNs were used to develop a dual-functional DDS for tumour theragnostics [102]. This approach utilised MSNs that were coated with a lipid bilayer for enhancing biocompatibility, followed by the addition of a paramagnetic lanthanide ion, gadolinium (GD), considering its T1 contrast for magnetic resonance. This carrier encapsulated a pro-apoptotic peptide (KLA), which successfully entered the cells and induced mitochondrial swelling and apoptosis.

Harini et al. reported in 2019 the use of polyethyleneimine (PEI)-modified MSNs as DDSs of curcumin to induce mitochondrial-mediated apoptosis in breast cancer (MCF-7) cells [103]. This approach focused on curcumin anti-cancer effects, as the latter was loaded into MSNs due to its hydrophobic nature and low solubility. However, despite the cytotoxic effects of PEI, it provided the carrier with numerous characteristics, such as the proton sponge effect that aided in endosomal escape, efficient drug uptake, and intracellular drug release. The novel carrier induced apoptosis by disrupting the mitochondria and nucleus.

#### Different Variations of Porous Silica/Carriers

Even though mesoporous silica are amongst the most common carriers used for targeting and drug delivery, several variations have been reported in the literature. Macroporous silica nanoparticles were developed to encapsulate a Bcl-2 converting peptide for the treatment of multidrug-resistant cancer cells [104]. The authors used a peptide derived from orphan nuclear receptor Nur77, which was loaded into the macroporous nanoparticles and managed to penetrate the mitochondria’s cytomembrane to bind with Bcl-2. Another example of using macroporous silica nanoparticles as a DDS of Bcl-2 converting peptide has been reported in 2020 [105]. This work focused on using folic acid as a targeting moiety for cancer cells while encapsulating N9 as the therapeutic peptide. These functionalised macroporous particles were internalised by HeLa cells and co-localised with the mitochondria while delivering N9 to induce 52% of HeLa cells towards apoptosis.

In 2022, Wang et al. reported the use of mesoporous carbon nanoparticles as potential nanocarriers to target the mitochondria [106]. Their approach was based on functionalising the carbon nanoparticles with chitosan and folic acid, considering the latter’s affinity to folate receptors and the API as M27-39. This novel carrier targeted the cancerous cell mitochondria and interfered with the mitochondrial energy metabolism process.

## Conclusion

In conclusion, using drug delivery systems to target the mitochondria remains a significant challenge due to various biological barriers, including cellular uptake limitations, mitochondrial membranes, and physiological processes that eliminate nanoparticles before they reach their target. However, advances in nanoparticle-based drug delivery systems, particularly porous carriers, offer promising solutions. These drug delivery systems, with their tuneable pore size, large surface area, and ease of functionalisation, provide versatile platforms for enhancing mitochondrial targeting. Techniques such as surface modifications with polyethylene glycol (PEG), the use of targeting ligands like triphenylphosphonium, and controlled, triggered drug delivery through pore capping have demonstrated potential for overcoming the barriers to effective mitochondrial drug delivery. Additionally, the integration of photodynamic therapy and reactive oxygen species (ROS) generation further boosts the effectiveness of these systems. Recent developments in functionalised porous silica and carbon nanoparticles also present exciting opportunities for more precise and efficient drug delivery mechanisms. This review underscores the importance of continued exploration and optimisation of porous carriers, especially silica-based systems, for mitochondrial targeting. These innovations are crucial in advancing mitochondrial therapies, providing a pathway for improved treatment strategies for mitochondrial-related diseases and conditions. As research in this area progresses, the potential for clinical applications continues to grow, making porous carriers a critical focus for future studies.

## Abbreviations

α-TOS, α-Tocopheryl succinate; API, active pharmaceutical ingredient; ATP, adenosine triphosphate; CPPs, cell penetrating peptides; DAMPs, danger-associated molecular patterns; DDS, drug delivery system; DOX, doxorubicin; ELVIS, inflammatory cell-mediated sequestration; EPR, enhanced permeability and retention; GD, gadolinium; HA, hyaluronic acid; ICG, indocyanine green; IMM, inner mitochondrial membrane; IMS, intermembrane space; M41S, Molecular 41 Sieves; MPPs, mitochondrial penetrating peptides; MSN, mesoporous silica nanoparticles; MTS, mitochondrial targeting sequences; NIR, near-infrared; OMM, outer mitochondrial membrane; PBA, phenylboronic acid; PDT, photodynamic therapy; PEG, polyethylene glycol; PEI, polyethyleneimine; PLD, poly L-aspartic acid; RES, reticuloendothelial system; RONS, reactive oxygen and nitrogen species; ROS, reactive oxygen species; SBA 15, Santa Barbara 15; SSPs, Szeto–Schiller peptides; TCA, tricarboxylic acid; TCPP, tumour-targeting cellular membrane-penetrating peptide; TPP^+^, Triphenylphosphonium.
